# Supplementary material for: Cancer in Migrants: A Population-Based Study in Italy
Source: Cancers (Basel). 2023 Jun 8;15(12):3103. doi: 10.3390/cancers15123103 (PMC10295978; doi:10.3390/cancers15123103)
Supplement: Supplementary file 1 [file cancers-15-03103-s001.zip › cancers-2324895-supplementary.pdf]

Supplementary materials.

Supplementary Table S1. Odds ratio of selected neoplasms among migrants, by region of origin.

Males

Cancer	Total	Sum	PMR	Interval
Head and neck	55	49,18618323	1,118200201	0,85-1,45
Colorectum	144	162,1714855	0,887948948	0,73-1,02
Lymphoma	87	89,65916893	0,970341361	0,78-1,19
Leukemia	76	77,71586429	0,977921312	0,78-1,22
Sarcoma	26	22,33324785	1,164183561	0,78-1,68
Stomach	46	45,80853641	1,004179649	0,74-1,33
Lung	194	174,4042878	1,112357973	0,96-1,28
Prostate	165	177,9897362	0,927019746	0,79-1,08
Bladder	173	163,0024609	1,06133367	0,91-1,23
Liver	44	44,3858448	0,99130703	0,73-1,32

Females

Cancer	Total	Sum	PMR	Interval
Head and neck	26	23,50145131	1,106314655	0,74-1,60
Colorectum	217	235,1204	0,9229314	0,81-1,05
Lymphoma	95	111,2716242	0,853766634	0,69-1,04
Leukemia	79	102,738387	0,768943355	0,61-0,95
Sarcoma	24	23,20185591	1,034400011	0,68-1,52
Cervix	165	61,84508904	2,667956382	2,28-3,10
Stomach	48	49,40054656	0,971649169	0,72-1,28
Lung	137	104,2700176	1,313896393	1,11-1,55
Breast	790	847,7103435	0,931922096	0,87-1,00
Bladder	65	61,42549793	1,05819248	0,82-1,34
Liver	37	29,82446829	1,240592108	0,89-1,69

Total population

Cancer	Total	Sum	PMR	Interval
Head and neck	81	72,68763454	1,114357353	0,89-1,38
Colorectum	361	397,2918855	0,908651833	0,82-1,01
Lymphoma	182	200,9307931	0,90578451	0,78-1,05
Leukemia	155	180,4542513	0,858943466	0,73-1,01
Sarcoma	50	45,53510376	1,098053938	0,82-1,44
Cervix	165	61,84508904	2,667956382	2,28-3,10
Stomach	94	95,20908297	0,98730076	0,80-1,20
Lung	331	278,6743054	1,187766484	1,07-1,32
Breast	790	847,7103435	0,931922096	0,87-1,00
Prostate	165	177,9897362	0,927019746	0,79-1,08
Bladder	238	224,4279588	1,060473932	0,93-1,20
Liver	81	74,21031309	1,091492498	0,87-1,35

1: EU, W/N Europe

Males

Cancer	Total	Sum	PMR	Interval
Head and neck	24	15,13572197	1,585652805	1,02-2,29
Colorectum	39	44,97069537	0,86723142	0,63-1,17
Lymphoma	29	31,29087908	0,926787641	0,63-1,31
Leukemia	14	23,76816833	0,589023092	0,34-0,96
Sarcoma	10	7,721128621	1,295147444	0,66-2,31

Females

Cancer	Total	Sum	PMR	Interval
Head and neck	16	9,867722395	1,621448128	0,96-2,58
Colorectum	88	95,48645348	0,921596696	0,74-1,13
Lymphoma	40	49,74481328	0,804103933	0,58-1,10
Leukemia	32	43,805515	0,730501627	0,51-1,02
Sarcoma	5	10,47446831	0,477351198	0,18-1,06

Total population

Cancer	Total	Sum	PMR	Interval
Head and neck	40	25,00344437	1,599779591	1,16-2,16
Colorectum	127	140,4571489	0,90419036	0,76-1,07
Lymphoma	69	81,03569236	0,851476652	0,67-1,07
Leukemia	46	67,57368333	0,680738384	0,50-0,90
Sarcoma	15	18,19559693	0,824375263	0,48-1,33

Stomach	14	13,03871631	1,073725332	0,61-1,76
Lung	61	45,95347537	1,327429525	1,02-1,70
Prostate	48	42,23063058	1,136615754	0,85-1,49
Bladder	48	44,56092431	1,077176938	0,80-1,42
Liver	10	12,19647187	0,819909241	0,42-1,46

Cervix	69	28,14212781	2,45184019	1,92-3,09
Stomach	16	21,87425932	0,731453338	0,43-1,16
Lung	61	43,09396023	1,415511586	1,09-1,81
Breast	338	371,8830734	0,908887831	0,82-1,01
Bladder	40	25,14782838	1,590594599	1,15-2,15
Liver	16	11,68969456	1,368726952	0,81-2,18

Cervix	69	28,14212781	2,45184019	1,92-3,09
Stomach	30	34,91297563	0,859279378	0,59-1,21
Lung	122	89,0474356	1,370056298	1,14-1,63
Breast	338	371,8830734	0,908887831	0,82-1,01
Prostate	48	42,23063058	1,136615754	0,85-1,49
Bladder	88	69,7087527	1,262395275	1,01-1,54
Liver	26	23,88616643	1,088496142	0,72-1,57

2: Balkans, E Europe

Males

Cancer	Total	Sum	PMR	Interval
Head and neck	14	6,101710413	2,294438617	1,31-3,76
Colorectum	15	15,84232624	0,946830647	0,55-1,53
Lymphoma	8	11,11022022	0,720057735	0,33-1,37
Leukemia	10	8,650346084	1,156023112	0,59-2,06
Sarcoma	5	2,567230426	1,94762416	0,71-4,31
Stomach	8	4,581265146	1,746242521	0,81-3,32
Lung	19	16,58451771	1,145646821	0,71-1,76
Prostate	5	14,05598444	0,355720371	0,13-0,79
Bladder	16	15,93038773	1,004369779	0,59-1,60
Liver	3	4,601574281	0,651950793	0,17-1,78

Females

Cancer	Total	Sum	PMR	Interval
Head and neck	1	4,817153087	0,207591493	0,01-1,02
Colorectum	38	41,72590834	0,910705159	0,65-1,24
Lymphoma	19	24,68003489	0,769853045	0,48-1,18
Leukemia	11	19,39529825	0,567147762	0,30-0,99
Sarcoma	7	5,058936638	1,383689993	0,61-2,74
Cervix	75	14,3258459	5,235292946	4,15-6,52
Stomach	10	9,522280302	1,050168624	0,53-1,87
Lung	31	20,67209975	1,499605767	1,04-2,10
Breast	153	185,8553885	0,823220684	0,70-0,96
Bladder	12	11,37786674	1,054679253	0,57-1,79
Liver	4	4,193927317	0,953759972	0,30-2,30

Total population

Cancer	Total	Sum	PMR	Interval
Head and neck	15	10,9188635	1,373769349	0,80-2,22
Colorectum	53	57,56823458	0,92064661	0,70-1,20
Lymphoma	27	35,79025511	0,754395293	0,51-1,08
Leukemia	21	28,04564433	0,748779374	0,48-1,13
Sarcoma	12	7,626167064	1,573529651	0,85-2,67
Cervix	75	14,3258459	5,235292946	4,15-6,53
Stomach	18	14,10354545	1,276274825	1,78-1,98
Lung	50	37,25661746	1,342043465	1,01-1,76
Breast	153	185,8553885	0,823220684	0,70-0,96
Prostate	5	14,05598444	0,355720371	0,13-0,79
Bladder	28	27,30825447	1,025331005	0,69-1,46
Liver	7	8,795501598	0,795861376	0,34-1,57

3: N Africa

Males

Cancer	Total	Sum	PMR	Interval
Head and neck	8	13,56498658	0,589753624	0,27-1,12
Colorectum	56	57,17744353	0,979407202	0,75-1,26
Lymphoma	20	17,18286418	1,163950305	0,73-1,77

Females

Cancer	Total	Sum	PMR	Interval
Head and neck	4	3,176341232	1,259310542	0,40-3,03
Colorectum	40	39,86032755	1,003504047	0,73-1,35
Lymphoma	15	12,3365479	1,215899303	0,71-1,96

Total population

Cancer	Total	Sum	PMR	Interval
Head and neck	12	16,74132781	0,716789023	0,38-1,21
Colorectum	96	97,03777108	0,989305493	0,80-1,20
Lymphoma	35	29,51941209	1,18566047	0,83-1,63

Leukemia	16	20,36748897	0,785565664	0,47-1,25
Sarcoma	4	3,99115466	1,002216236	0,32-2,42
Stomach	14	15,50246411	0,903082239	0,51-1,48
Lung	72	66,98519277	1,074864414	0,85-1,35
Prostate	71	79,74873435	0,890296261	0,70-1,12
Bladder	76	58,44186943	1,300437524	1,03-1,62
Liver	14	15,55979903	0,899754552	0,51-1,47

Leukemia	11	15,12310696	0,727363764	0,38-1,27
Sarcoma	5	2,461425955	2,031342844	0,74-4,51
Cervix	6	5,47746609	1,095397014	0,44-2,28
Stomach	11	8,601474074	1,278850567	0,67-2,22
Lung	14	15,37922864	0,910318738	0,52-1,49
Breast	87	91,76830702	0,948039719	0,76-1,16
Bladder	7	9,805385505	0,7138934	0,31-1,41
Liver	9	6,428953171	1,39991687	0,68-2,57

Leukemia	27	35,49059593	0,760764909	0,51-1,09
Sarcoma	9	6,452580615	1,394790788	0,68-2,56
Cervix	6	5,47746609	1,095397014	0,44-2,28
Stomach	25	24,10393819	1,037174913	0,68-1,50
Lung	86	82,36442141	1,044140158	0,84-1,28
Breast	87	91,76830702	0,948039719	0,76-1,16
Prostate	71	79,74873435	0,890296261	0,70-1,12
Bladder	83	68,24725493	1,216166131	0,95-1,50
Liver	23	21,9887522	1,045989322	0,67-1,54

4: Sub-Saharan Africa

Males

Cancer	Total	Sum	PMR	Interval
Head and neck	1	3,461293003	0,288909375	0,01-1,42
Colorectum	3	10,50696258	0,285524953	0,07-0,77
Lymphoma	10	9,826771394	1,017628232	0,51-1,81
Leukemia	10	7,116181784	1,405247969	0,70-2,50
Sarcoma	3	2,760605181	1,086718239	0,27-2,95
Stomach	3	2,931510588	1,023363181	0,26-2,78
Lung	11	11,1012098	0,990882994	0,52-1,73
Prostate	11	11,48130465	0,958079272	0,50-1,66
Bladder	7	10,61336637	0,659545686	0,29-1,34
Liver	8	2,911504508	2,747720286	1,27-5,22

Females

Cancer	Total	Sum	PMR	Interval
Head and neck	0	1,096242728	0	0,00-2,74
Colorectum	4	11,63694736	0,343732757	0,11-0,82
Lymphoma	5	4,852965723	1,030297819	0,37-2,28
Leukemia	4	4,571460157	0,874993954	0,27-2,11
Sarcoma	1	0,962466966	1,038996698	0,05-5,13
Cervix	2	2,448204882	0,816925093	0,13-2,70
Stomach	2	0,332235645	6,01982368	1,01-20,02
Lung	8	5,017138812	1,594534315	0,74-3,03
Breast	45	36,33197181	1,238578524	0,91-1,64
Bladder	2	3,005293211	0,665492469	0,11-2,19
Liver	0	1,606729382	0	0,00-1,87

Total population

Cancer	Total	Sum	PMR	Interval
Head and neck	1	4,557535731	0,219416821	0,01-1,08
Colorectum	7	22,14390994	0,316114002	0,13-0,62
Lymphoma	15	14,67973712	1,021816663	0,59-1,64
Leukemia	14	11,68764194	1,197846415	0,68-1,96
Sarcoma	4	3,723072147	1,074381543	0,34-2,59
Cervix	2	2,448204882	0,816925093	0,13-2,70
Stomach	5	3,263746233	1,531981852	0,56-3,4
Lung	19	16,11834861	1,178780808	0,73-1,80
Breast	45	36,33197181	1,238578524	0,91-1,64
Prostate	11	11,48130465	0,958079272	0,50-1,66
Bladder	9	13,61865958	0,66085799	0,32-1,21
Liver	8	4,51823389	1,770603336	0,82-3,36

5: W Asia

Males

Cancer	Total	Sum	PMR	Interval
Head and neck	0	0,277639035	0	0,00-11,09

Females

Cancer	Total	Sum	PMR	Interval
Head and neck	0	0,081299107	0	0,00-37,44

Total population

Cancer	Total	Sum	PMR	Interval
Head and neck	0	0,358938142	0	0,00-8,55

Colorectum	1	1,368275913	0,730846747	0,03-3,62
Lymphoma	0	0,391307027	0	0,00-7,68
Leukemia	2	1,244136225	1,607541007	0,27-5,32
Sarcoma	0	0,2809417	0	0,00-10,69
Stomach	0	0,366110131	0	0,00-8,32
Lung	0	1,597317481	0	0,00-1,88
Prostate	3	1,845404079	1,625660219	0,41-4,43
Bladder	3	1,406064716	2,133614453	0,54-5,83
Liver	0	0,426944105	0	0,00-7,13

Colorectum	1	1,077070028	0,928444738	0,04-4,60
Lymphoma	1	0,275375674	3,631402827	0,18-18,27
Leukemia	0	0,373035601	0	0,00-8,09
Sarcoma	0	0,05783321	0	0,00-59,91
Cervix	0	0,132844153	0	0,00-23,04
Stomach	0	0,24915299	0	0,00-12,48
Lung	0	0,357303294	0	0,00-8,55
Breast	1	2,181441	0,458412581	0,02-2,26
Bladder	1	0,255279168	3,917280075	0,20-19,73
Liver	0	0,183117355	0	0,00-16,64

Colorectum	2	2,44534594	0,817880189	0,13-2,70
Lymphoma	1	0,666682701	1,499963923	0,07-7,47
Leukemia	2	1,617171826	1,236726963	0,20-4,10
Sarcoma	0	0,33877491	0	0,00-9,07
Cervix	0	0,132844153	0	0,00-23,04
Stomach	0	0,61526312	0	0,00-4,49
Lung	0	1,954620775	0	0,00-1,53
Breast	1	2,181441	0,458412581	0,02-2,26
Prostate	3	1,845404079	1,625660219	0,41-4,43
Bladder	4	1,661343885	2,407689363	0,76-5,81
Liver	0	0,610061459	0	0,00-4,91

6: Other Asia excl. Japan

Males

Cancer	Total	Sum	PMR	Interval
Head and neck	4	3,200070704	1,249972382	0,39-3,01
Colorectum	8	8,040381494	0,994977664	0,46-1,88
Lymphoma	2	5,86501063	0,34100535	0,05-1,12
Leukemia	9	4,150605906	2,168358115	1,05-3,98
Sarcoma	2	1,376639571	1,452813098	0,24-4,82
Stomach	3	2,316398909	1,295113716	0,33-3,53
Lung	11	8,379048929	1,312798158	0,69-2,28
Prostate	4	7,250588799	0,551679334	0,17-1,33
Bladder	2	8,011953801	0,249627001	0,04-0,82
Liver	3	2,274502645	1,318969669	0,33-3,59

Females

Cancer	Total	Sum	PMR	Interval
Head and neck	1	0,897308078	1,114444441	0,05-5,54
Colorectum	11	7,804464544	1,409449673	0,74-2,45
Lymphoma	1	5,120319079	0,195300329	0,01-0,96
Leukemia	9	4,835039151	1,861412022	0,90-3,41
Sarcoma	2	1,144964892	1,746778451	0,29-5,79
Cervix	4	2,881128173	1,388345037	0,44-3,35
Stomach	3	0,145138369	20,66993045	5,45-58,32
Lung	3	3,906349577	0,767980423	0,19-2,09
Breast	34	37,71242185	0,901559707	0,63-1,24
Bladder	0	2,103930352	0	0,00-1,42
Liver	2	0,811858546	2,463483337	0,41-8,15

Total population

Cancer	Total	Sum	PMR	Interval
Head and neck	5	4,097378782	1,220292354	0,44-2,71
Colorectum	19	15,84484604	1,199128092	0,74-1,83
Lymphoma	3	10,98532971	0,273091485	0,06-0,74
Leukemia	18	8,985645057	2,003195083	1,22-3,10
Sarcoma	4	2,521604463	1,586291609	0,50-3,82
Cervix	4	2,881128173	1,388345037	0,44-3,35
Stomach	6	2,461537279	2,437501171	0,98-5,07
Lung	14	12,28539851	1,139564174	0,64-1,86
Breast	34	37,71242185	0,901559707	0,63-1,24
Prostate	4	7,250588799	0,551679334	0,17-1,33
Bladder	2	10,11588415	0,197708868	0,03-0,65
Liver	5	3,086361191	1,620030739	0,59-3,59

7: N America, Oceania, Japan

Males

Females

Total population

Cancer	Total	Sum	PMR	Interval
Head and neck	2	2,785480391	0,718009004	0,12-2,37
Colorectum	12	11,32186216	1,059896317	0,57-1,80
Lymphoma	7	5,086830116	1,376102571	0,60-2,72
Leukemia	6	5,778778853	1,038281643	0,42-2,16
Sarcoma	0	1,50679939	0	0,00-1,99
Stomach	2	3,374738889	0,592638443	0,09-1,96
Lung	8	10,664202	0,750173337	0,34-1,42
Prostate	11	10,18002072	1,080547899	0,56-1,87
Bladder	13	11,19600148	1,161128821	0,64-1,93
Liver	1	2,859016123	0,349770675	0,01-1,73

Cancer	Total	Sum	PMR	Interval
Head and neck	4	1,416737154	2,823388931	0,90-6,84
Colorectum	15	15,86579192	0,945430274	0,54-1,52
Lymphoma	7	5,331856493	1,312863542	0,57-2,59
Leukemia	7	6,042885246	1,158387048	0,50-2,29
Sarcoma	0	1,202354989	0	0,00-2,49
Cervix	2	3,295939611	0,606807234	0,10-2,01
Stomach	2	3,739251687	0,534866376	0,08-1,77
Lung	7	6,317601477	1,108015443	0,48-2,19
Breast	46	48,31340571	0,952116691	0,70-1,25
Bladder	0	4,071658387	0	0,00-0,73
Liver	4	2,17688261	1,837489988	0,58-4,44

Cancer	Total	Sum	PMR	
Head and neck	6	4,202217545	1,427817559	0,57-2,97
Colorectum	27	27,18765408	0,99309782	0,66-1,42
Lymphoma	14	10,41868661	1,343739429	0,76-2,20
Leukemia	13	11,8216641	1,099675976	0,61-1,83
Sarcoma	0	2,709154379	0	0,00-1,10
Cervix	2	3,295939611	0,606807234	0,10-2,01
Stomach	4	7,113990576	0,562272322	0,17-1,35
Lung	15	16,98180347	0,88329841	0,51-1,42
Breast	46	48,31340571	0,952116691	0,70-1,25
Prostate	11	10,18002072	1,080547899	0,56-1,87
Bladder	13	15,26765986	0,85147299	0,47-1,42
Liver	5	5,035898733	0,992871435	0,36-2,20

8: Latin America

Males

Cancer	Total	Sum	PMR	Interval
Head and neck	2	4,659281135	0,429250767	0,07-1,42
Colorectum	10	12,94353826	0,772586274	0,39-1,37
Lymphoma	11	8,905286286	1,235221378	0,64-2,14
Leukemia	9	6,64015814	1,355389406	0,66-2,48
Sarcoma	2	2,128748301	0,939519247	0,15-3,11
Stomach	2	3,697332323	0,540930548	0,09-1,79
Lung	12	13,13932374	0,913289012	0,49-1,55
Prostate	12	11,19706853	1,071709078	0,58-1,82
Bladder	8	12,84189303	0,622961115	0,28-1,18
Liver	5	3,556032235	1,406061495	0,51-3,12

Females

Cancer	Total	Sum	PMR	Interval
Head and neck	0	2,148647525	0	0,00-1,39
Colorectum	20	21,66343677	0,923214549	0,57-1,40
Lymphoma	7	8,929711108	0,78389994	0,34-1,55
Leukemia	5	8,592046645	0,581933526	0,21-1,29
Sarcoma	4	1,839404945	2,174616314	0,69-5,27
Cervix	7	5,14153242	1,361461803	0,59-2,69
Stomach	4	4,936754169	0,810248974	0,25-1,95
Lung	13	9,526335844	1,364638011	0,75-2,27
Breast	86	73,66433423	1,167457779	0,93-1,43
Bladder	3	5,65825618	0,530198687	0,13-1,44
Liver	2	2,733305356	0,731714807	0,12-2,42

Total population

Cancer	Total	Sum	PMR	Interval
Head and neck	2	6,80792866	0,293775111	0,04-0,97
Colorectum	30	34,60697503	0,866877269	0,59-1,22
Lymphoma	18	17,83499739	1,009251619	0,61-1,56
Leukemia	14	15,23220478	0,91910529	0,52-1,50
Sarcoma	6	3,968153245	1,512038379	0,61-3,15
Cervix	7	5,14153242	1,361461803	0,59-2,69
Stomach	6	8,634086492	0,694920071	0,28-1,44
Lung	25	22,66565958	1,102990182	0,72-1,60
Breast	86	73,66433423	1,167457779	0,93-1,43
Prostate	12	11,19706853	1,071709078	0,58-1,82
Bladder	11	18,50014921	0,594589799	0,31-1,03
Liver	7	6,289337591	1,112994795	0,48-2,20
